# Keep hospitals dry as much as much as possible in order to prevent infections

**DOI:** 10.25122/jml-2017-0063

**Published:** 2019

**Authors:** Ali Mehrabi Tavana

**Affiliations:** Health Management Research Center, Baqiyatallah University of Medical Sciences, Tehran, Iran

**Keywords:** dry hospitals, prevention, infections

Unfortunately, nowadays most hospitals around the world are infected [1], and hospital infections kill patients and sometimes even hospital personnel [2–3]. Also, there are too many costs to ward off infections in hospitals in order to prevent antibiotic resistance, and patients are more likely to suffer an infection [4]. Healthcare practitioners need a better environment in order to serve patients better, so these measurements are necessary and should be performed unquestionably. However, despite all the efforts, infections are still prevalent in hospitals, and the germs are becoming more resistant to antibiotics [5–9]. An instinctual question is whether there is a simpler solution and even though there are probably several solutions, the one that is currently practiced is a thorough disinfection practice of hospitals using different sanitizing products, adding moisture to the dry air. This environment of the hospitals provides the best development habitat for microorganisms, allowing them to develop abundantly. Physicians can transfer germs to patients during an examination, but patients could also spread infections to physicians and other healthcare practitioners. It seems to be better to keep the hospital environment dry as much as possible. However, during hospital transfer or severe cases such as sepsis, it is essential first to use detergent and to ensure the proper use of specific disinfectants and sterilization methods. It is of great significance to share experience in this regard [10], even though the general public is well aware of its importance in order to control the spreading of infections. Keeping the hospital environment dry can help prevent and control hospital infections. Of course, the use of disinfectants is highly recommended in some cases where the infection is confirmed by a piece of evidence or if it is likely to cause an hospital-acquired infection and affect the lives of patients. Mainly if microbial resistance is seen in the hospital or among doctors and other healthcare professionals, the use of disinfectants is unavoidable. Physicians, along with the hospital’s infection control team must take additional care of patients dealing with infections, the collaboration between them being essential.

## Conflict of Interest

The authors confirm that there are no conflicts of interest.
